# Characterization and evaluation of avermectin solid nanodispersion prepared by microprecipitation and lyophilisation techniques

**DOI:** 10.1371/journal.pone.0191742

**Published:** 2018-01-23

**Authors:** Bo Cui, Chunxin Wang, Xiang Zhao, Junwei Yao, Zhanghua Zeng, Yan Wang, Changjiao Sun, Guoqiang Liu, Haixin Cui

**Affiliations:** Institute of Environment and Sustainable Development in Agriculture, Chinese Academy of Agricultural Sciences, Beijing, China; VIT University, INDIA

## Abstract

Poorly water-soluble and photosensitive pesticide compounds are difficult to formulate as solvent-free nanoformulations with high efficacy. A avermectin solid nanodispersion with a mean particle size of 188 nm was developed by microprecipitation and lyophilisation techniques. The suspensibility and wetting time of the solid nanodispersion in water were 99.8% and 13 s, respectively, superior to those of conventional water dispersible granules and wettable powders. The anti-photolysis performance of the nanoformulation was twice that of the technical material, and the biological activity against diamondback moths was more than 1.5 times that of the conventional solid formulations while taking LC 50 as the evaluation index. Moreover, the formulation composition substantially decreased the surfactant content and avoided organic solvents. Microprecipitation combined with lyophilisation is an easy and promising method to construct solid nanoformulations for pesticides with poor water solubility and environmental sensitivity. The application of the highly effective solid nanodispersion in crop production will have a great potential in reducing chemical residues and environmental pollution.

## Introduction

Pesticides have been widely used as a kind of primary agrochemical to control weeds, pests and plant diseases for ensuring and improving crop yields. However, 70 to 90 percent of the applied pesticides are lost or decomposed due to the climate conditions and administration methods [1–3]. In addition, most pesticide active ingredients are poorly soluble in water which limits the development of their highly effective formulations. The low efficacy of pesticides further results in their overuse and a series of food safety and environmental pollution issues.

Avermectin, a biological insecticide, is a macrocyclic lactone compound with strong activity against a broad spectrum of pests combined with low toxicity to non-target organisms [4–6]. However, the low water solubility and instability to light seriously influence its bioavailability and shelf life [7]. Currently, the main commercial formulations of avermectin include emulsifiable concentrate (EC), wettable powder (WP), water dispersible granule (WDG) and aqueous capsule suspension (CS) [8–11]. The conventional EC, WP and WDG have disadvantages such as overdosage of organic solvent and surfactants, dust drift and low efficacy. Though a microcapsule is capable of protecting photosensitive compounds against degradation by capsulation, the complexity of production processes, incomplete release of active ingredients, high cost and low degradation rate of the capsule wall materials still hinder its application in agriculture [11,12].

Nanotechnology has provided a new approach to construct novel pesticide formulations and improve their performance by nanosizing. As described by the Ostwald-Freundlich and Noyes-Whitney equations, the solubility and dissolution rate of materials increase with decreasing particle size. Therefore, the water dispersibility of the formulations with particle size at the nanoscale can be improved compared with conventional formulations [13]. Furthermore, the increased specific surface area induced by size reduction is also beneficial to enhance the spreading, coverage and retention of pesticide particles on the surface of crop leaves and targeted organisms. Accordingly, the stronger bioavailability of active ingredients can be achieved [8].

During the production of nanoformulations, top-down and bottom-up techniques have been developed. Among the top-down methods, wet-milling and high-pressure homogenization have been widely used to prepare pharmaceutical nanosuspensions for drug delivery [14–17]. For this kind of method, specialized equipment and high energy input are needed. Moreover, the in-process heat generation may damage the physicochemical properties of the heat-sensitive pesticides and decrease their biological activity. Compared with the top-down approach, microprecipitation as a bottom-up technique constructs nanoparticles from the molecular state. The procedure is simple and can be controlled more precisely, so it facilitates scaling up and application to most pesticide compounds with low solubility in water. In our previous studies, the lambda-cyhalothrin and chlorantraniliprole nanoformulations have been produced by high- pressure homogenization [18,19]. Nevertheless, the relevant research about microprecipitation in the construction of pesticide nanoformulations is still rarely reported.

In the present research, microprecipitation combined with the lyophilisation technique was successfully applied to prepare an avermectin solid nanodispersion. This method is applicable to poorly water-soluble pesticides with certain solubility in organic solvents, especially to the sensitive compounds in the environment. The particle size, interfacial charge, crystallinity, suspensibility, wettability, stability and bioavailability have been characterized to evaluate the formulation performances. This investigation has provided a promising strategy to construct highly effective nanoformulations for sensitive pesticides. This solid nanodispersion could substantially reduce the surfactant dosage and decrease the frequency of administration relative to conventional formulations. It has a significant application prospect for crop and environmental protections.

## Materials and methods

### 2.1. Materials

Avermectin technical material (TC, 95%) was obtained from Qilu Pharmaceutical (Inner Mongolia) Co., Ltd. (Inner Mongolia, China). 1-Dodecanesulfonic acid sodium salt (SDS), sodium dodecylbenzenesulfonate (SDBS), sodium lauryl ether sulfate (SLES), polyoxyethylene sorbitan monooleate (Tween 80), sorbitan monooleate (Span 80), polyvinylpyrrolidone K30 (PVP K30), 2-(2-hydroxy-5-tert-octylphenyl)benzotriazole (UV 329), ethyl acetate, methanol and sucrose were purchased from J&K Scientific Ltd. (Beijing, China). Hydroxypropyl methylcellulose (HPMC) was supplied by Sigma-Aldrich Shanghai Trading Co., Ltd. (Shanghai, China). Maleic rosin-polyoxypropylene-polyoxyethylene ether sulfonate (MRES) and polycarboxylate were provided by Sinvochem S&D Co., Ltd. (Jiangsu, China). The Kaiwei (10%, w/w) and Cuiwei (10%, w/w) WDGs of avermectin were purchased from Beijing Huarong Biological Hormone Plant (Beijing, China) and Beijing Anda Hexin Sci-Tech Development Co., Ltd. (Beijing, China), respectively. The Yipaohong WP (1.8%, w/w) was purchased from Shandong Luobang Biological Pesticide Co., Ltd. (Shandong, China). The Qiantou WP (1.8%, w/w) was bought from Shandong Rongbang Chemical Co., Ltd. (Shandong, China). The standard hard water (Ca^2+^ + Mg^2+^ = 342 mg/l) was obtained from China Agricultural University. All the chemicals were used as received.

### 2.2. Preparation of the avermectin solid nanodispersion

The avermectin solid nanodispersion was prepared by microprecipitation followed by freeze-drying. Among the composition of the avermectin solid nanodispersion, the avermectin and light stabilizer UV 329 were poorly soluble in water, so they were dissolved in ethyl acetate. In contrast, the amphiphilic surfactants MRES and polycarboxylate were easier to dissolve in water than in organic solvent. Therefore, the preparation process was as follows. First, 4.2 g avermectin technical material (equivalent to 4.0 g avermectin active ingredient) and 0.4 g UV 329 were dissolved in 27.8 ml ethyl acetate to obtain the organic phase. Second, 0.4 g MRES and 0.4 g polycarboxylate were dissolved in a 7.5% (w/w) ethyl acetate aqueous solution containing 5.8 ml ethyl acetate and 64.6 ml water. The organic phase was then added to the aqueous solution dropwise while stirring at 800 rpm for 15 minutes on a magnetic stirrer (RCT Basic, IKA^®^-Works Guangzhou, Guangzhou, China). The mixture was emulsified at 10000 rpm for 15 minutes by a shearing machine (C25, ATS Engineering Ltd., Vancouver, Canada) to acquire the aqueous dispersion. Subsequently, 34.6 g sucrose as an antifreeze agent and water-soluble carrier was added into the prepared dispersion while stirring at 800 rpm for 15 minutes on a magnetic stirrer (RCT Basic, IKA^®^-Works Guangzhou, Guangzhou, China). Finally, the dispersion was freeze-dried for 48 h using a freeze drier (FD-81, EYELA, Tokyo, Japan) to convert the liquid into the solid powder.

### 2.3. Particle size and zeta potential measurements

The mean particle size, polydispersity index (PDI) and zeta potential of the samples were characterized at 25°C using a Zetasizer Nano ZS 90 (Malvern, Worcestershire, UK). All particle sizes and PDIs of the samples were measured by dynamic light scattering (DLS) in triplicate and the data were recorded as the mean ± standard deviation (S.D.).

### 2.4. Morphological and structural characterizations of particles

The morphological characterization of the avermectin technical material was performed using a scanning electron microscope (SEM, JSM-7401F, JEOL, Tokyo, Japan). We dropped 3 μl of the aqueous dispersion onto a freshly cleaned silicon slice. The sample was air-dried and coated with platinum for 40 s by a sputter coater (ETD-800, Beijing Elaborate Technology Development Ltd., Beijing, China). The image was recorded at 3 kV and the work distance (WD) was 6.6 mm.

The morphology of the nanoparticles was characterized by a transmission electron microscope (TEM, H-7650, HITACHI, Tokyo, Japan). We dropped 3 μl of the sample on a carbon film supported on a copper grid. The grid was left overnight under ambient conditions for complete dryness. The accelerating voltage of TEM imaging was 80 kV.

X-ray diffraction (XRD) was applied to confirm the crystallinity of the samples by a diffractometer (D8 ADVANCE, Bruker AXS Inc., Karlsruhe, Germany) using CuKα radiation. The measurement conditions were as follows: tube voltage of 40 kV, tube current of 40 mA, step scan mode with a step size of 2θ = 0.02°, and counting time of 0.1s per step.

### 2.5. Determination of the avermectin content

The avermectin content was analysed by high-performance liquid chromatography (HPLC, 1260 Infinity, Agilent, California, USA) using a C18 column (5 um, 4.6 mm*150 mm, Agilent, California, USA) at room temperature. The mobile phase was composed of methanol and water (90:10, v/v) which has been used in other studies for the quantification of avermectin [20–22]. The flow rate was 0.5 ml/min and the UV detector wavelength was 245 nm. Milli-Q water (18.2 MΩ.cm, TOC ≤ 4 ppb) was used for the preparation of all the solutions in this measurement.

### 2.6. Suspensibility test

The suspensibility was tested and calculated according to CIPAC MT 184. The samples were added slowly to a beaker containing 50 ml standard hard water (30 ± 1°C). After swirling by hand in a circular motion at a rate of approximately 120 times per minute for 2 minutes, the suspension was placed in a 30 ± 1°C constant temperature bath for 13 minutes. The solution was then transferred to a 250-ml measuring cylinder. Subsequently, 200 ml standard hard water was used to rinse the beaker and fill the cylinder to scale. The measuring cylinder was then stoppered and inverted 30 times by hand and placed in the 30°C water bath in an upright position free from vibration. After standing for 30 minutes, the top 225 ml of the solution was removed. The pesticide contents of the original suspension and the remaining 25 ml of solution were measured by HPLC. The value was calculated by the following equation:

$$\text{Suspensibility}(\%)=\frac{10}{9}\times \frac{\text{m}1-\text{m}2}{\text{m}1}\times 100$$

Here, m_1_ (mg) and m_2_ (mg) are the pesticide contents of the original suspension and of the left 25-ml solution at the bottom, respectively.

### 2.7. Wettability test

The wettability was measured according to CIPAC MT 53. The 100 ml standard hard water was added into a 250-ml beaker that was placed in a water bath at 25 ± 1°C. When the temperature of the standard hard water reached 25°C, 5.000 g sample was poured onto the water surface at once. Immediately, the time was recorded with a stopwatch until the sample was entirely wetted by water. The average value of three tests was adopted.

### 2.8. Photolysis test

The photolytic behaviour of avermectin in the solid nanodispersion was evaluated with the same amount of avermectin technical material as a control. The samples were dissolved in methanol and transferred into culture dishes. After the methanol volatilized completely, the samples were exposed to a 400 W xenon lamp as simulated sunlight at a distance of 20 cm in the photolysis test chamber (XT5409-XPC80, Xutemp Temptech Co., Ltd., Zhejiang, China). At specified irradiation time intervals at 0 h, 24 h, 48 h, 72 h, 96 h, 120 h, 144 h, 168 h, 192 h, 216 h, 240 h and 264 h, the samples were removed. The avermectin in the culture dish was fully extracted with methanol, and the content was analysed by HPLC.

### 2.9. Bioassays

Bioassays were conducted using the leaf-dip method according to NY/T 1154.14–2008. The avermectin solid nanodispersion, Cuiwei WDG, Kaiwei WDG and Yipaohong WP were directly diluted with pure water to different concentrations based on the concentration of the active ingredient. Then, cabbage (*Brassica oleracea* L.) leaves were immersed in the above dispersions for 10 s. Afterwards, the leaves were air-dried and placed in a culture dish with a filter paper. Ten second-instar diamondback moth (*Plutella xylostella* L.) larvae were introduced into each dish, and three replications were carried out. The bioassays to diamondback moth (*Plutella xylostella* L.) were usually conducted with second-instar or third-instar larvae. In contrast, the second-instar larvae may be more sensitive to pesticide than the third-instar larvae. In the experiment, the identification of second-instar larvae was based on their unique morphological characteristics according to GB/T 23392.3–2009: body length of 2.0–3.0 mm, head width of 0.244 mm, black head, body color from gray to canary yellow. The head is wider than body and the pronotum has two discontinuous U-shaped patterns. Mortality was assessed after treatment for 48 h. Concentration-mortality data were analysed using DPS 8.1 (Refine Information Technology Co., Ltd., Hangzhou, China).

### 2.10. Statistical analysis

The data were analysed by one-way analysis of variance (ANOVA) and Duncan’s multiple range test with the software package SPSS^®^. Results with a probability (P) of less than 0.05 were deemed to be statistically significant.

## Results and discussion

### 3.1. Surfactant optimization

The avermectin solid nanodispersion was prepared by freeze-drying the avermectin aqueous dispersion that was produced by microprecipitation. As reported previously [23], the properties of the solid nanodispersion significantly depended on the particle size and distribution of the pre-prepared aqueous dispersion, so the composition and content of surfactants in the nanoformulation were optimized using the mean particle size and PDI of the aqueous dispersion as evaluation indices.

Surfactant plays a vital role in decreasing the interfacial tension, stabilizing formed emulsions and hindering particle aggregation, leading to particle size reduction. During the preparation of the avermectin aqueous dispersions, the ethyl acetate solution with avermectin and UV 329 was added into the 7.5% (w/w) ethyl acetate aqueous solution containing surfactants. The solubility of ethyl acetate in water is approximately 80 g/L (20°C). However, the content of ethyl acetate in the mixture reached 469 g/L, which was much higher than the saturation solubility of ethyl acetate in water. In this condition, the undissolved ethyl acetate was suspended in water as oil droplets, and an oil-water interface between water and ethyl acetate formed. The amphiphilic surfactants could adsorb on the oil-water interface to keep the system stable. The commonly used surfactants in the pesticide formulation composition involve anionic and nonionic type surfactants. As shown in Table 1, the aqueous dispersions containing 4.0% (w/w) avermectin, 0.8% (w/w) single surfactant and 0.4% (w/w) UV 329 as a light stabilizer were prepared by microprecipitation. The effects of five anionic (SDS, SDBS, polycarboxylate, MRES and SLES) and four nonionic (tween 80, span 80, PVP K30 and HPMC) surfactants on the particle size and distribution of the dispersions were compared. Among the nine surfactants, the nanoparticles stabilized with single MRES exhibited the smallest size and narrow size distribution compared to the other systems. Therefore, MRES was fixed to combine with the other surfactants in a 1:1 (w/w) ratio to construct the avermectin aqueous dispersions.

**Table 1:** Effect of a single surfactant on the particle size and dispersibility of the avermectin aqueous dispersions.

surfactant^a^	mean size (nm) ± S.D.	PDI^b^ ± S.D.^c^
SDS	588 ± 43 de	0.283 ± 0.224 a
SDBS	480 ± 29 e	0.239 ± 0.207 a
Polycarboxylate	426 ± 36 e	0.264 ± 0.194 a
MRES	265 ± 11 e	0.254 ± 0.026 a
SLES	2317 ± 186 b	0.584 ± 0.391 a
Tween 80	392 ± 5 e	0.351 ± 0.040 a
Span 80	939 ± 74 d	0.694 ± 0.531 a
PVP K30	4820 ± 568 a	0.514 ± 0.243 a
HPMC	1592 ± 272 c	0.663 ± 0.430 a

^a^SDS: 1-dodecanesulfonic acid sodium salt; SDBS: sodium dodecylbenzenesulfonate; MRES: maleic rosin-polyoxypropylene-polyoxyethylene ether sulfonate; SLES: sodium lauryl ether sulfate; Tween 80: polyoxyethylene sorbitan monooleate; Span 80: sorbitan monooleate; PVP K30: polyvinylpyrrolidone K30; HPMC: hydroxypropyl methylcellulose.

^b^PDI: polydispersity index.

^c^S.D.: standard deviation of three measurements.

Different letters indicate significant differences according to Duncan’s multiple range test at P < 0.05.

Table 2 shows the particle size and dispersibility of the aqueous dispersions with 4.0% (w/w) avermectin, 0.8% (w/w) composite surfactant and 0.4% (w/w) UV 329. The combination of polycarboxylate and MRES at the ratio of 1:1 (w/w) reduced the particle size to 46 nm, which was obviously smaller than the others. This result demonstrates that the combination of polycarboxylate and MRES indeed presented better performance than single surfactant. The -COO^−^ group on the polycarboxylate molecular skeleton and -SO_3_^−^ group of MRES may interact with the -OH group of the avermectin molecule through hydrogen bonds [24,25]. Both the hydrogen bonding and Van der Waals force between avermectin and the two anionic surfactants could make the surfactants adsorb on the pesticide surface. Accordingly, the anionic polycarboxylate and MRES made the pesticide particles negatively charged, and they repelled each other to prevent the formation of large clusters. Furthermore, the long hydrophobic chains of the two polymers could also induce steric hindrance against aggregation. Both polycarboxylate and MRES were capable of providing electrostatic repulsion and steric stabilization effects, so this combination was chosen and the content of the composite surfactant was further optimized.

**Table 2:** Effect of composite surfactants on the particle size and dispersibility of the avermectin aqueous dispersions.

surfactant^a^	mean size (nm) ± S.D.	PDI^b^ ± S.D.^c^
MRES + SDS	222 ± 4 b	0.253 ± 0.018 b
MRES + SDBS	240 ± 4 b	0.328 ± 0.026 a
MRES + Polycarboxylate	46 ± 1 c	0.350 ± 0.046 a
MRES + SLES	241 ± 1 b	0.251 ± 0.014 b
MRES + Tween 80	226 ± 4 b	0.218 ± 0.006 b
MRES + Span 80	212 ± 3 b	0.202 ± 0.027 b
MRES + PVP K30	226 ± 4 b	0.218 ± 0.021 b
MRES + HPMC	431 ± 52 a	0.059 ± 0.043 c

^a^SDS: 1-dodecanesulfonic acid sodium salt; SDBS: sodium dodecylbenzenesulfonate; MRES: maleic rosin-polyoxypropylene-polyoxyethylene ether sulfonate; SLES: sodium lauryl ether sulfate; Tween 80: polyoxyethylene sorbitan monooleate; Span 80: sorbitan monooleate; PVP K30: polyvinylpyrrolidone K30; HPMC: hydroxypropyl methylcellulose.

^b^PDI: polydispersity index.

^c^S.D.: standard deviation of three measurements.

Different letters indicate significant differences according to Duncan’s multiple range test at P < 0.05.

**Fig 1:**
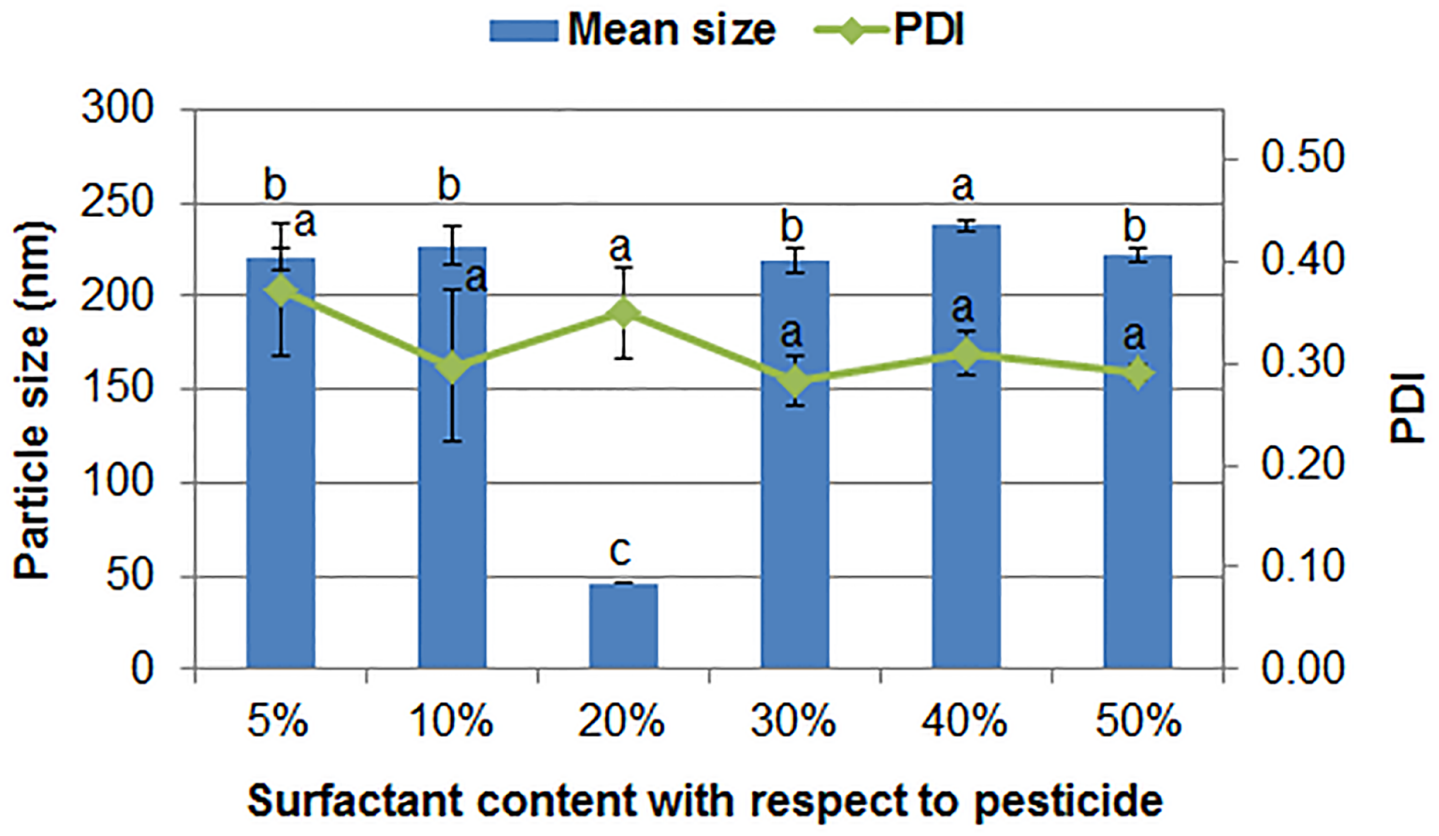
Particle size and dispersibility of the aqueous dispersions containing 4.0% (w/w) avermectin with different surfactant contents. Different letters indicate significant differences according to Duncan’s multiple range test at P < 0.05.

The surfactant content affects the wettability and stability of the pesticide particles, and further influences the re-dispersibility and bioavailability of the solid formulation. Taking the 1:1 (w/w) mixture of polycarboxylate and MRES as a composite surfactant, the effect of surfactant content on the particle size and dispersibility of the avermectin dispersion was shown in Fig 1. The pesticide nanoparticles exhibited the smallest size when the surfactants accounted for 20% (w/w) of the avermectin content. When the surfactant amount was not enough to provide efficient electrostatic repulsion and steric hindrance effects, the neighbouring nanoparticles may approach and aggregate. However, the excessive surfactants may cause interface layer of particles thickening, accompanied by an increase in particle size [26]. Meanwhile, the significant entanglement of polymer chains may also lead to the formation of agglomerates by intermolecular hydrophobic interactions [27,28]. Then, the composite surfactant with a 1:1 (w/w) ratio of polycarboxylate to MRES and 20% of pesticide content were determined to prepare the avermectin solid nanodispersion.

Both polycarboxylate and MRES are anionic polymer surfactants. Polycarboxylate has been widely used as a kind of pesticide and detergent additive, and its toxicity has also been reported. Three linear polycarboxylate compounds, including two linear polyacrylates (90000 MW and 4500 MW) and one linear polyacrylate-maleate copolymer (12000 MW), were tested for their teratogenic potential in female Sprague Dawley rats. The minimum no-effect dose was 1125 mg/kg/day for the 90000 MW polyacrylate, 3000 mg/kg/day for the 4500 MW polyacrylate and 6670 mg/kg/day for the polyacrylate-maleate copolymer. This research clarified that these polycarboxylates were not developmentally toxic, even at very high dose levels [29]. Moreover, sodium polyacrylate, one of the common polycarboxylates, has been approved as a food additive by the Food and Drug Administration (FDA) and China [30]. MRES has a similar structure with alkylethoxysulfates. According to the previous report, the no-observed-effect-concentration of alkylethoxysulfate (C_13.5_EO_3_S) for *Lepomis macrochirus*, *Daphnia magna* and *Desmodesmus subspicatus* were 2.2 mg/L, 0.34 mg/L and 0.9 mg/L, respectively [31]. Considering the 3–6 g/ hm^2^ dosage of the avermectin active ingredient against diamondback moth (*Plutella xylostella* L.) and cabbage worm (*Pieris rapae* L.) in China [32], the usage amount of single polycarboxylate and MRES while applying the solid nanodispersion is approximately 0.6 mg/L. This is a relatively low and safe dosage.

The water-based formulations tend to break down over time due to gravitational separation, flocculation and Ostwald ripening [33]. Therefore, to improve the stability and prolong the shelf life of the formulation, the aqueous dispersion was freeze-dried into solid powder after adding sucrose. As an antifreeze agent and water-soluble carrier, sucrose can not only protect dispersion from freezing and desiccation impairment but can also accelerate redispersion of the solid nanodispersion [34]. Furthermore, it can improve the suspensibility and stability of the re-dispersed dispersion by increasing its viscosity [35].

### 3.2. Characterization and evaluation of the solid nanodispersion

#### 3.2.1 Size and morphology

**Fig 2:**
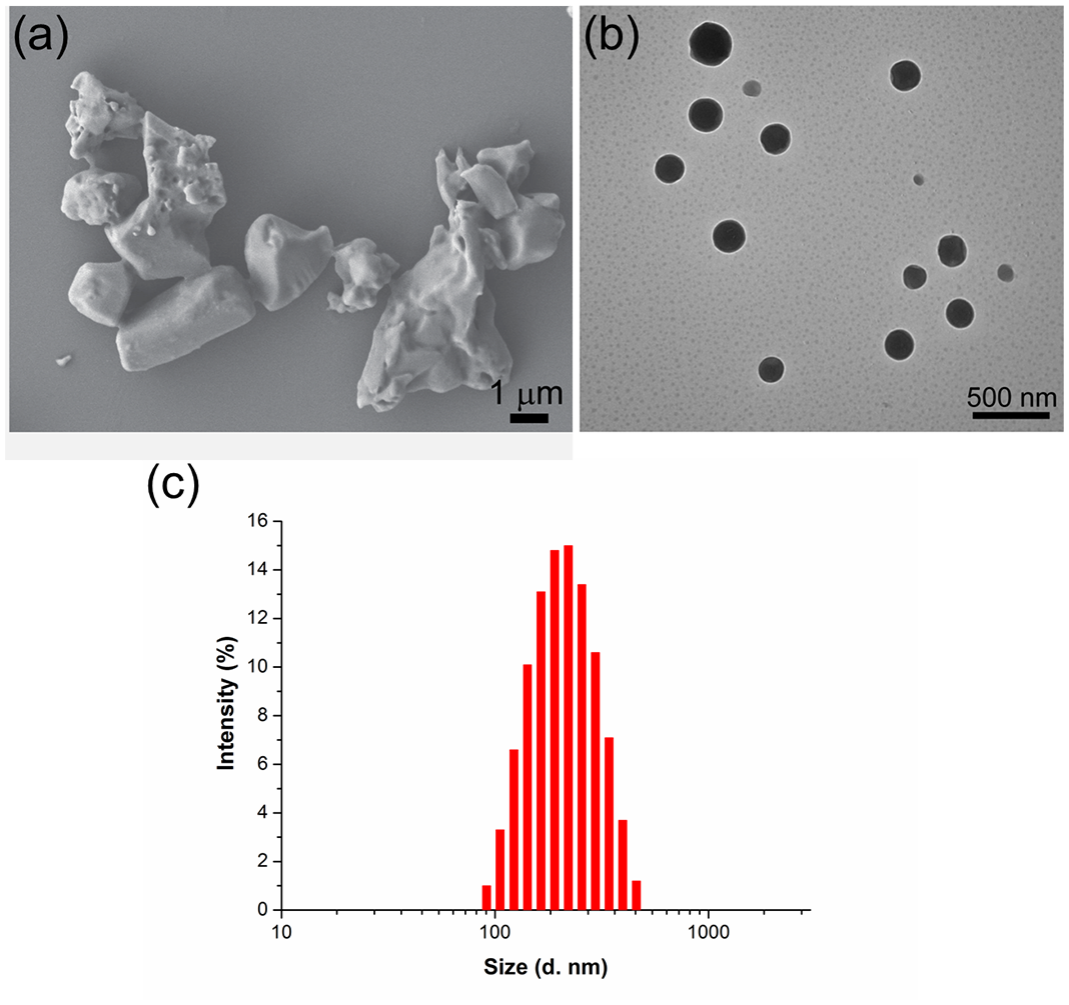
The morphology and particle size of the avermectin TC and nanoparticles. (a) SEM image of TC with magnification of 8000; (b) TEM image of the nanoparticles with magnification of 40000; (c) Size of the nanoparticles measured by DLS. Size (d. nm): diameter of the nanoparticles.

As shown in Fig 2a, the particles of the avermectin TC after dispersing by water presented an irregular blocky structure with a micrometer size because of its poor water solubility and dispersibility. In the following, the solid nanodispersion containing 10% (w/w) avermectin, 2% (w/w) composite surfactants, 1% (w/w) UV 329 and 87% (w/w) sucrose produced by solidifying the avermectin aqueous dispersion was evaluated in detail. In the formulation composition, the content of the surfactants accounted for 20% (w/w) of the active ingredient. In addition, the composition of the solid nanodispersion was free of solvent, so a nanosuspension yielded when the powder was freely re-dispersed in water. As observed from the TEM image (Fig 2b), the avermectin nanoparticles appeared as spherical shapes, and the size was mainly in the range of 105 nm to 210 nm. The mean size and PDI of the nanoparticles measured by DLS were 188 ± 8 nm and 0.292 ± 0.143, respectively (Fig 2c), consistent with the result of TEM. The particle size of the solid powder was larger than that of the pre-prepared aqueous dispersion shown in Fig 1 because the particle aggregation during lyophilisation was difficult to avoid. A similar phenomenon has also been reported in other nanoformulations [13,36]. The above results demonstrate that this nanosizing method could effectively construct the avermectin solid nanodispersion.

#### 3.2.2. Zeta potential and pH

Zeta potential and pH are the typical indicators of the stability of water-based formulations [37]. It is generally believed that an aqueous dispersion with an absolute zeta potential higher than 30 mV has long-term stability [38]. However, when the surfactants could provide steric stabilization in addition to electrostatic repulsion, the value usually decreases due to the shear plane shifting to a larger distance from the particle surface [39–42]. In this research, the zeta potential of the re-dispersed nanosuspension was—33 mV, predicting an excellent physical stability. The negative charge revealed that the anionic polymers covered the surface of the neutrally charged avermectin [43]. In addition, the pH of the nanosuspension was measured to be 7.0. The neutral condition was beneficial to avoid the decomposition of the sensitive active ingredient.

#### 3.2.3. Crystallinity

**Fig 3:**
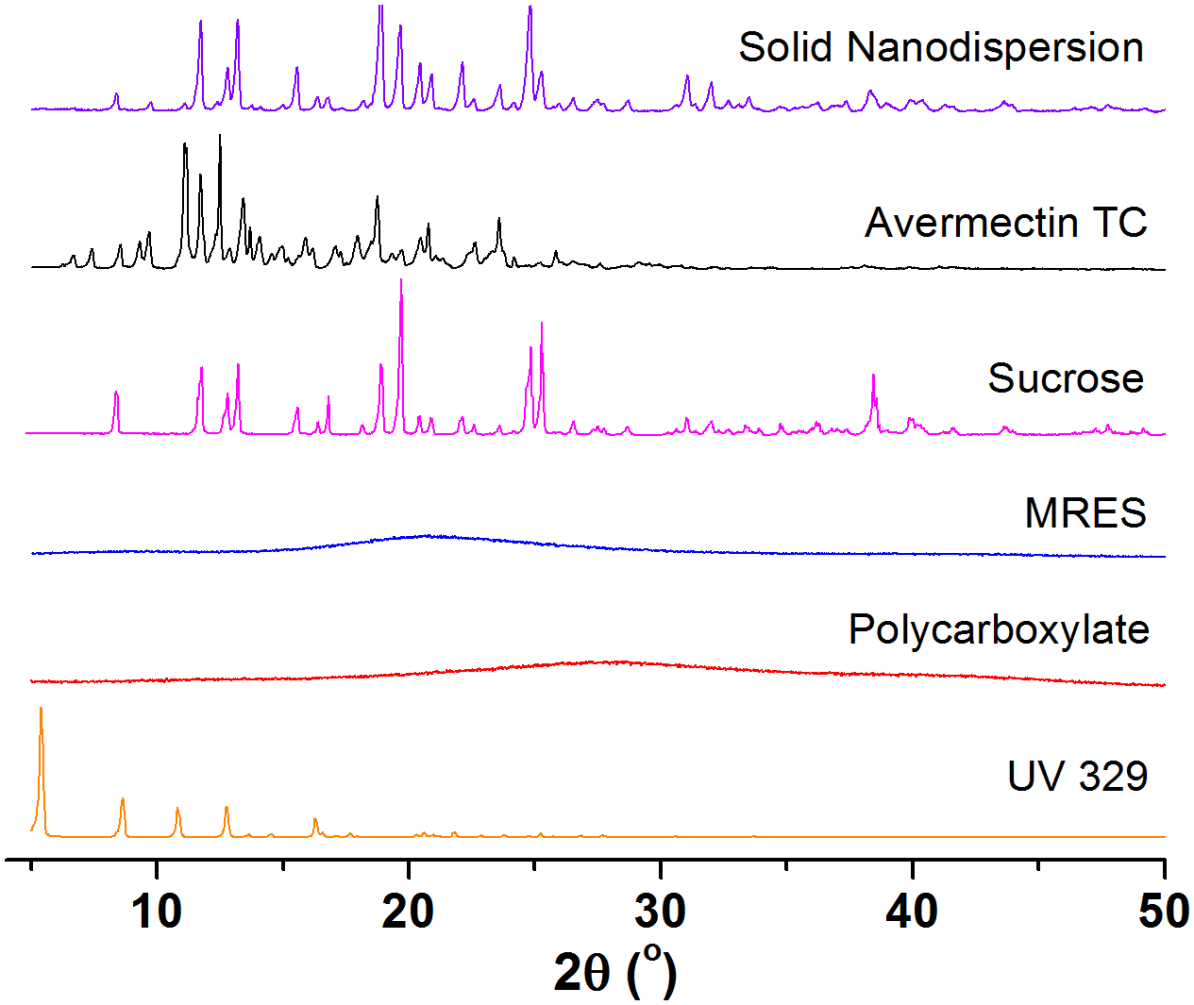
XRD patterns of the avermectin solid nanodispersion and pure components in the formulation.

As shown in Fig 3, the avermectin TC, solid nanodispersion, sucrose and UV 329 presented crystalline characteristics, but polycarboxylate and MRES surfactants showed an amorphous state. By comparison, although the intense peaks of the solid nanodispersion mainly derived from sucrose crystal, the characteristic peaks of avermectin at 9.7°, 11.1°, 12.5° and 15.0° could also been observed. The above result suggests that the crystal structure of avermectin has been preserved during the nanosizing process. According to the literatures [44,45], the crystalline state could improve stability during storage compared with the amorphous form, which increases molecular mobility and leads to aggregation.

#### 3.2.4. Suspensibility and wettability

Suspensibility and wettability are important indices of the re-dispersibility in water and spreadability on leaves of solid formulations. These characteristics significantly affect the pesticide efficacy by influencing pesticide retention on targeted organisms. According to CIPAC MT 184, the suspensibilities of four avermectin commercial solid formulations, including Kaiwei WDG, Cuiwei WDG, Qiantou WP and Yipaohong WP, were measured to be 96.7%, 90.3%, 97.2% and 27.5%, respectively. In contrast, the suspensibility of the avermectin solid nanodispersion was 99.8%, higher than those of the conventional WP and WDG formulations. It has been reported that suspensibility is in inverse proportion to particle size, because Brownian motion may dominate the gravitational force when the particle size decreases [46,47]. Furthermore, sucrose could increase solution viscosity and reduce particle sedimentation rate.

The wettability tests of the solid powder in water were carried out according to CIPAC MT 53 in the same condition. The wetting times of the avermectin solid nanodispersion and two WPs (Qiantou and Yipaohong) were 13 s, 19 s and 22 s, respectively. The shorter wetting time reveals that the wettability of the nanoformulation was superior to that of the conventional pesticide formulations. The enlarged specific surface area of nanoparticles was conducive to enhancing their contact area with water and shortening the wetting time. In addition, the water-soluble carrier sucrose further accelerated the dispersion speed of the particles. In conclusion, the excellent re-dispersibility of the solid nanodispersion could attribute to its small size effect and formulation composition.

#### 3.2.5. Storage stability

**Fig 4:**
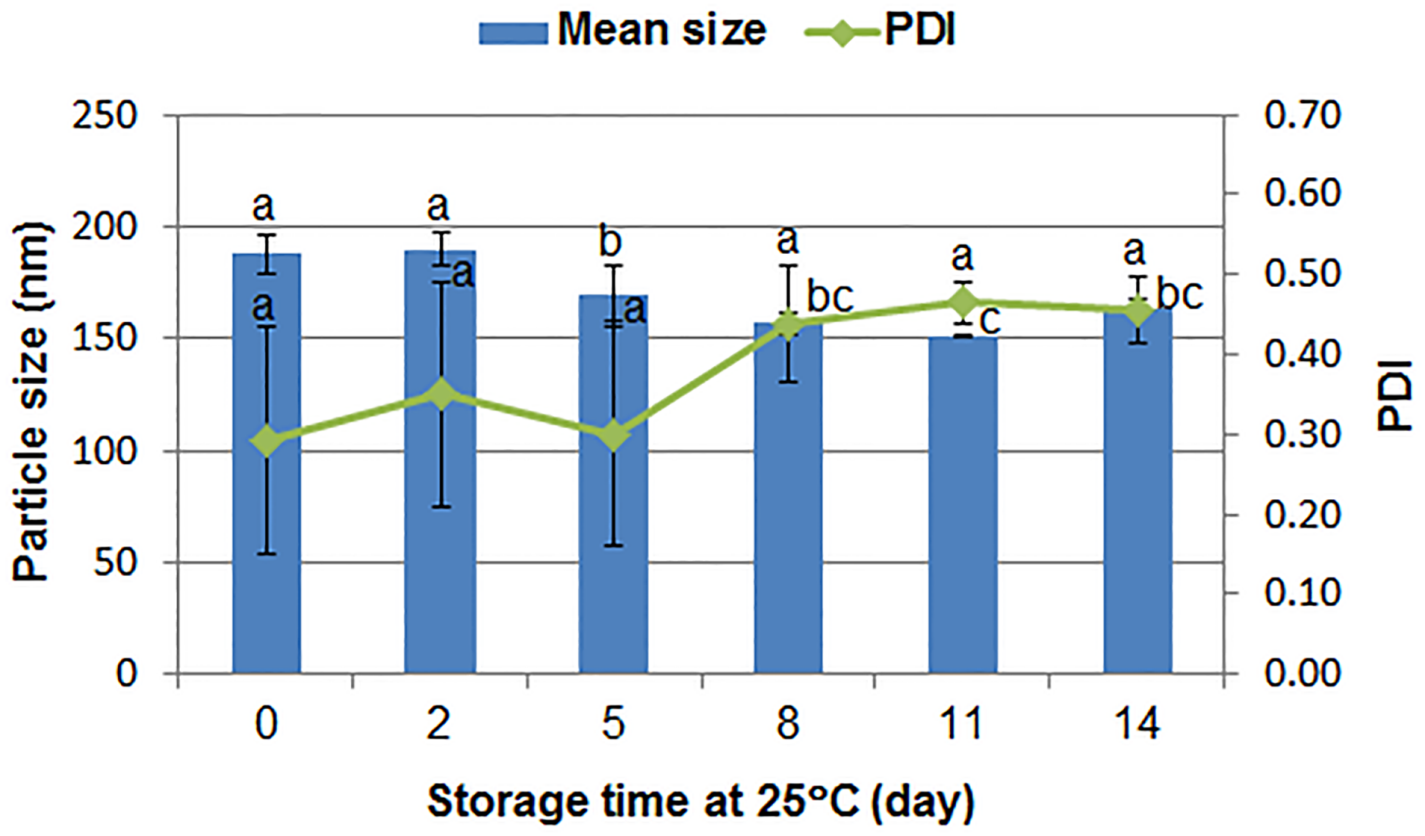
Stability of the avermectin solid nanodispersion at 25°C. Different letters indicate significant differences according to Duncan’s multiple range test at P < 0.05.

Fig 4 shows the storage stability of the avermectin solid nanodispersion tested at 25°C. The mean size of the nanoparticles changed slightly and was kept at around 180 nm during storage at 25°C for 14 days. Though the PDI increased from 0.292 to 0.439 within eight days, the data remained nearly the same in subsequent measurements. Polycarboxylate and MRES surfactants are liquid at room temperature, which may promote molecular motion. However, the content of the surfactants only accounted for 2% (w/w) of the composition. Most of the component in the nanoformulation was sucrose which was stable during storage. Moreover, the crystalline structure and narrow size distribution of the solid nanodispersion could effectively prevent particle coalescent and recrystallization, prolonging its shelf life.

#### 3.2.6. Photolysis property

**Fig 5:**
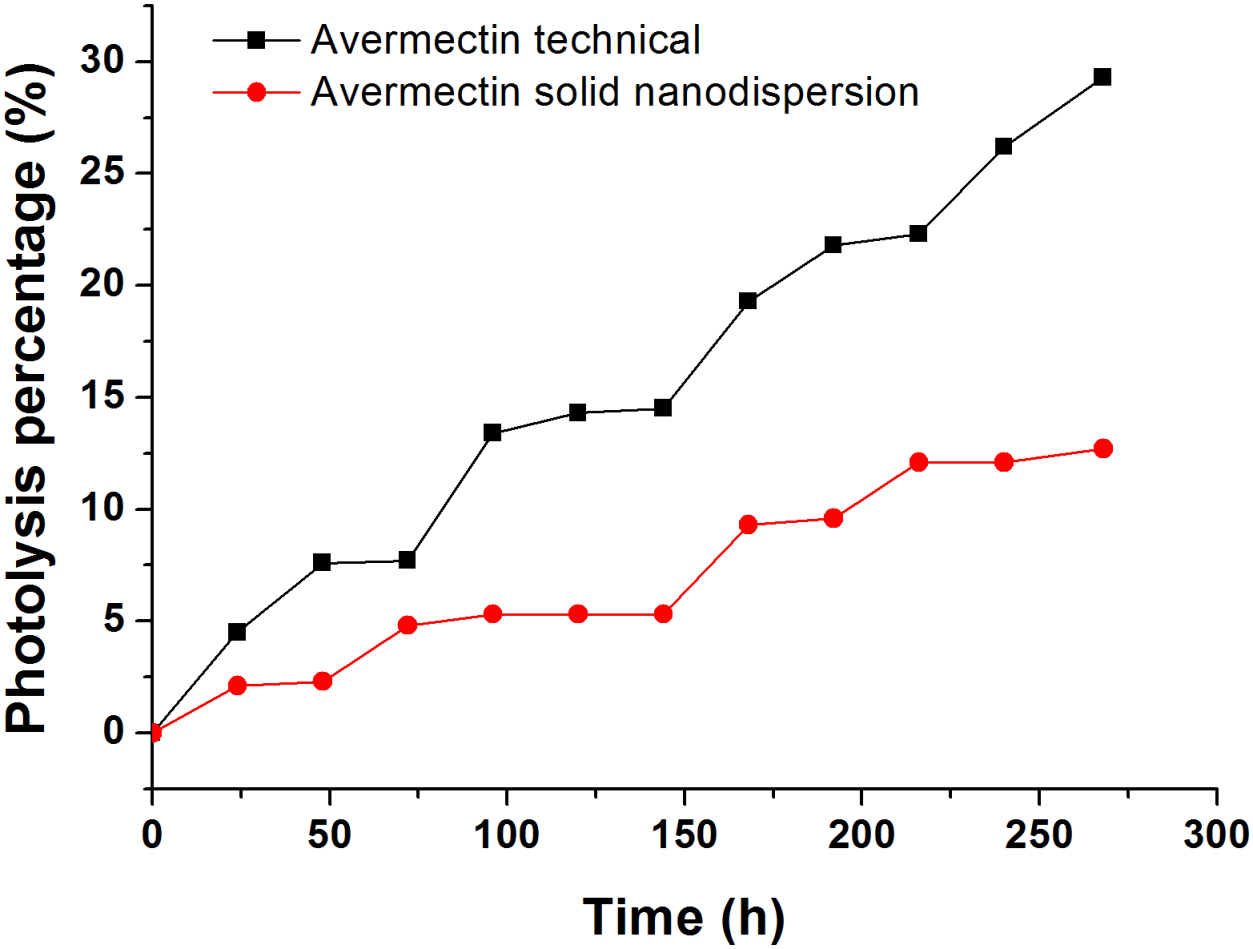
Photolysis of avermectin in the solid nanodispersion and technical material.

Avermectin is sensitive to UV light and undergoes photodegradation in the presence of light. UV 329 is a kind of benzotriazole UV stabilizer that is widely used to protect materials against UV-radiation [48–50]. It possesses a phenolic group attached to a benzotriazole structure and has excellent absorption capacity with a full spectrum of UV light [51]. In addition to UV 329, the carrier in the formulation composition could also provide a UV shielding effect for avermectin. The photolytic behaviours of the solid nanodispersion and pure avermectin are presented in Fig 5. After 264 hours of exposure under simulated light, the photolysis percentage of avermectin in the solid nanodispersion was only 13%. In contrast, the free avermectin degraded 29%, more than twice higher than the solid nanodispersion. The slow decomposition rate of the nanoformulation was due to the protection of UV 329 and the sucrose carrier to the pesticide compound. Though the microcapsule structure has been intensively studied to protect the photosensitive avermectin [52,53], it is difficult to completely exert the effect of the active ingredient encapsulated in the shell. In this investigation, the solid nanodispersion could be adequately dispersed in water and release pesticide to avoid losses. The enhanced UV shielding property of the avermectin solid nanodispersion predicts its improved photostability which could extend the pesticide efficacy period and decrease the frequency of spraying.

#### 3.2.7. Biological activity

The bioassay results of the avermectin solid nanodispersion and three conventional formulations to diamondback moths (*Plutella xylostella* L.) were listed in Table 3. Taking LC 50 as the evaluation index, the toxicity of the solid nanodispersion was 3.4, 1.5 and 14.2 times that of Cuiwei WDG, Kaiwei WDG and Yipaohong WP, respectively. In contrast, the toxicity of the nanoformulation was 2.2, 1.3 and 44.9 times that of Cuiwei WDG, Kaiwei WDG and Yipaohong WP while using LC 90 as the evaluation parameter. It has also been demonstrated that particle size reduction could induce higher bioavailability in the lambda-cyhalothrin nanoformulation system [23]. The improvement of the formulation performances in dispersibility, wettability and photostability jointly promoted the enhancement of the pesticide biological activity [54]. This highly effective nanoformulation could dramatically decrease pesticide dosage and totally avoid residue of organic solvents, improving its environmental friendliness.

**Table 3:** Bioassay results of four avermectin formulations.

Formulation	Toxicity regression equation	Correlation coefficient	LC 50^a^ (μg/mL)	95% confidence limit of LC 50	LC 90^b^ (μg/mL)	95% confidence limit of LC 90
Cuiwei WDG^c^	Y = 2.2448+1.3173x	0.9872	43.71	32.0~51.6	162.74	92.8–285.4
Kaiwei WDG	Y = 1.9092+2.5132x	0.9836	20.07	13.3~30.2	94.14	58.0–152.7
Yipaohong WP^d^	Y = 1.0118+2.6934x	0.9992	184.43	159.2~213.6	3347.63	2333.5–4802.6
Solid Nanodispersion	Y = 1.6857+3.1245x	0.9664	12.96	7.9~21.3	74.61	35.6–156.4

^a^LC 50: median lethal concentration.

^b^LC 90: 90% lethal concentration.

^c^WDG: water dispersible granule.

^d^WP: wettable powder.

## Conclusion

In this research, the solid nanodispersion of poorly water-soluble and photolabile avermectin was prepared by microprecipitation and lyophilisation techniques. This method could dramatically reduce production costs compared to the commonly used wet-milling and high-pressure homogenization approaches and is suitable for constructing solid nanoformulations for most sensitive pesticides in the environment. The avermectin solid nanodispersion with mean particle size of 188 nm presented improved formulation characteristics in dispersibility, wettability, anti-photolysis and biological activity compared to the conventional pesticide formulations. Therefore, the application of this nanoformulation in crop protection has broad prospects for enhancing pesticide efficacy and reducing chemical residues in agricultural products and the environment.
